# Esophageal Duplication Cyst Treated Thoracoscopically During the Neonatal Period: Clinical Case Report

**DOI:** 10.1097/MD.0000000000002270

**Published:** 2015-12-11

**Authors:** Barbara Cuch, Pawel Nachulewicz, Andrzej Pawel Wieczorek, Magdalena Wozniak, Elzbieta Pac-Kozuchowska

**Affiliations:** From the Department of Paediatric Surgery and Traumatology (BC, PN), Department of Paediatric Radiology (APW, MW), and Department of Paediatric, Medical University of Lublin, Lublin, Poland (EP-K).

## Abstract

Esophageal duplication cysts (EDCs) are rare developmental anomalies. They may occur anywhere along the esophagus with the predominant location in the thoracic segment. Presently, most are diagnosed prenatally or in early childhood. The prevalence of EDCs is estimated at 1 in 8200 live births. Usually, cysts are asymptomatic in the neonatal period, but they may cause respiratory distress or feeding difficulties depending on the size and location of the lesion.

This report presents a female neonate with a cyst located in the right pleural cavity recognized prenatally. Computed tomography confirmed the diagnosis and revealed a round cystic mass in proximity to the left lung base. Thoracoscopic cyst excision was undertaken on day 15 after delivery. The postoperative period was uneventful. Histological cyst examination confirmed the diagnosis of foregut duplication.

This case underlines the importance of early diagnosis and treatment of EDC, before symptoms and complications arise, and confirms that surgery in the neonatal period is safe and effective.

## INTRODUCTION

Esophageal duplication cysts (EDCs) are an uncommon congenital anomaly, and account for 20% of gastrointestinal duplications. The etiology of this entity is explained by several theories.^1–3^ According to Bentley and Smith, gastrointestinal duplications occur as a consequence of a split notochord. This anomaly seems to result in impaired development of the vertebrae, spinal cord, muscles, and gastrointestinal tract. In 1944, Bremer proposed a theory of faulty recanalization during the solid stage of development in the foregut. Another explanation for gastrointestinal duplications was suggested by Lewis and Thyng, who claimed that these duplications are persistent gut diverticula, which normally disappear prenatally.^4^ EDCs are commonly divided into 3 types: cystic, tubular, and diverticular.^5^ Most of them elongate in the lower part of the thoracic esophagus, although cervical and abdominal cysts have been reported as well.^6^ The majority of patients are asymptomatic and the lesions are usually found incidentally. However, respiratory distress, dysphagia, retrosternal pain, hemoptysis, and infection can occur in the case of large cysts with rapid growth or an unfortunate location.^3^ Early surgical excision is always recommended to prevent a symptomatic course of EDC. Technical progress in surgical instrumentation with its minimization allows for the use of minimally invasive techniques in the treatment of this mediastinal cyst. Classical thoracotomy has been slowly squeezed out by video-assisted thoracoscopy and the age of patients treated successfully with this approach has diminished. Here, we report thoracoscopic excision of an EDC performed on a neonate on the 15th day after birth.

## CASE PRESENTATION

**FIGURE 1:**
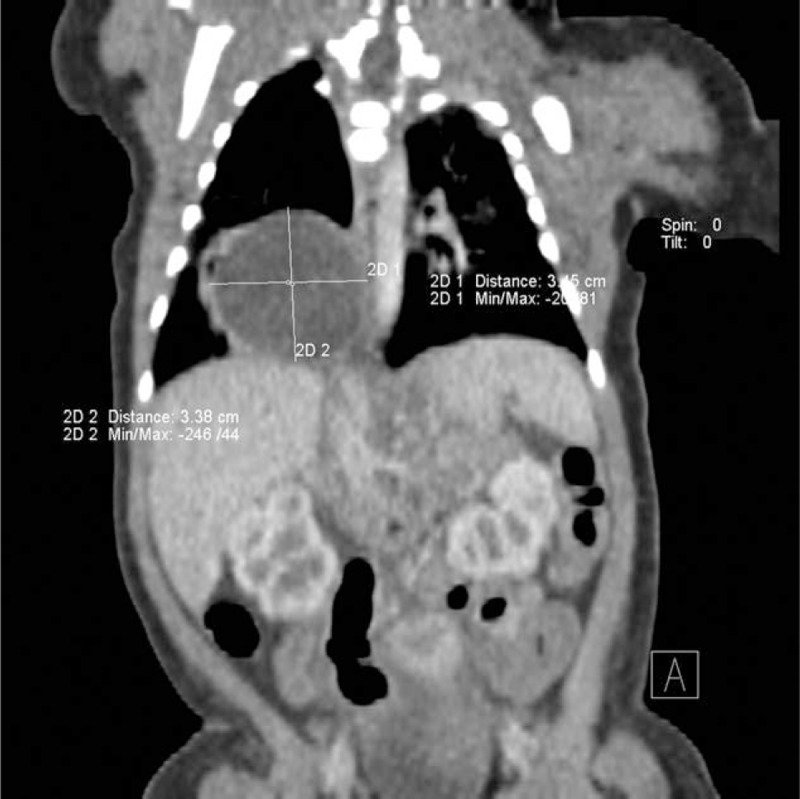
Computed tomography scan. Esophageal duplication cyst with the size fixed (frontal view).

**FIGURE 2:**
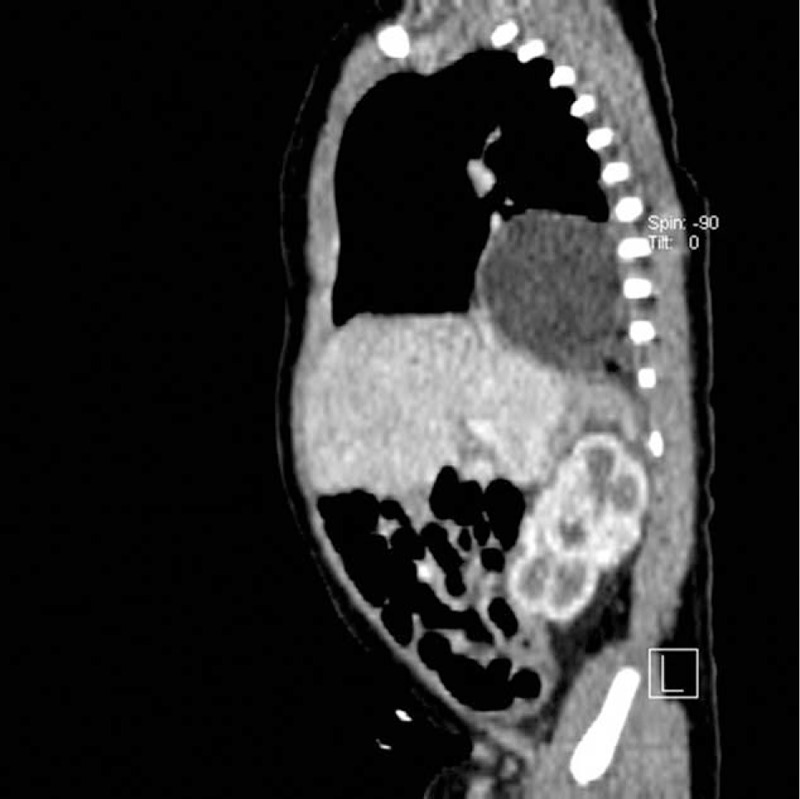
Computed tomography scan showed the well circumscribed mass in the posterior mediastinum (sagittal view).

The female neonate weighed 3770 g with 10 Apgar points in the 37 weeks of gestation and was admitted to the Department of Pediatric Surgery and Traumatology in Lublin on the first day of life due to a congenital thoracic cystic mass discovered by prenatal ultrasonography. Upon admission, the neonate was in general good condition. Chest and abdominal radiographs revealed a round mass in the right lung field. On the second day after birth, a chest computed tomography (CT) scan was performed. It showed a cystic mass measuring 35 mm in proximity to right lung base (Figs. 1 and 2).

The initial diagnosis of duplication of the esophagus or a bronchogenic cyst was made. The elevated bilirubin level decreased significantly during a period of 2 weeks and on the 15th day after birth, thoracoscopy was performed.

The patient was in an almost prone position with a slight elevation of the right side of the thorax. The first 3 mm camera port was inserted below the tip of the scapula and 5 mm positive pressure insufflation was started. The second 3 mm port was located paravertebrally and the camera was changed to this port. Under direct visualization, the third 3 mm port was inserted at the anterior auxiliary line. The large cystic mass filled almost the entire pleural cavity, so initial puncture was necessary. The lesion was connected to the inferior part of the esophagus and was excised using a hook with electrocoagulation. The continuity of the esophagus was confirmed by pumping the air through the esophageal tube while the esophagus at the site of resection was submerged in a 0.9% saline solution. The thoracic tube was left in the right pleural cavity. Postoperative recovery was uneventful. A control chest radiograph was performed and the thoracic tube was removed on the fourth postoperative day. The excised mass was a unilocular cyst with a smooth thick wall. Histological examination showed boundless smooth muscle cells with a collagenous stroma within the cyst wall. The internal surface was lined with a ciliated epithelium. Based on these data, a final diagnosis of a foregut duplication cyst was established. On the seventh postoperative day, the patient was discharged in general good condition. The infant remains symptom-free 1 year after surgery. We got the parental consent to use clinical data of their child for scientific purposes (it was a routine consent which parents are asked for on admission to our hospital).

## DISCUSSION

EDCs are rare congenital malformation. The incidence is estimated to be 1 in 8200 live births with male prevalence. EDCs mainly elongate in the thoracic segment of the esophagus, accounting for 30% of posterior mediastinal masses in children. A duplication in the cervical or abdominal part of the esophagus is also possible, but this occurs less frequently.^7^ Diagnosis of EDC can be challenging because of its rarity and difficult differential diagnosis. It has to be distinguished from many mediastinal masses such as bronchogenic cysts, mature cystic teratomas, pericardial cysts, congenital cystic adenomatoid malformations, and neurogenic tumors.^8^ These mediastinal masses may have similar appearance by ultrasonography, x-ray and even CT visualization.^9^ Magnetic resonance imagination (MRI) is the most sensitive and relatively precise tool to make the diagnosis. It can reveal the characteristic structure of a cyst with a thin wall and fluid content and exclude concomitant anomalies such as spina bifida.^7,8^ In our case, there was no possibility of performing MRI, so we based our diagnosis on a CT chest scan.

EDCs are often an incidental finding during routine radiological investigations.^1^ Most of patients are asymptomatic prenatally and after birth, although 80% of them develop symptoms during childhood.^10^ Cough, stridor, feeding difficulties, and chest pain may occur, depending on the size, location, and growth of the EDC. Some cases of cardiac arrhythmia and bleeding from the cyst with an ectopic gastric mucosa have been reported.^1,9^ In more than 80% of patients, diagnosis is established before the age of 2 years, when the symptoms usually appear.^4^ Early diagnosis and treatment seem to be crucial for an asymptomatic course of the entity. In recent years, most mediastinal masses have been diagnosed prenatally, although a differential diagnosis with some similar lesions remains difficult in this evaluation. Some studies have suggested prenatal magnetic resonance imaging to further differentiate cystic mediastinal lesions revealed in prenatal ultrasound examinations.^1^ However, a histological examination is ultimately required to confirm the clinical diagnosis. There are 3 main criteria of an EDC: (1) it must be attached to or within the esophageal wall, (2) it is covered by 2 muscle layers, and (3) it is lined with a squamous, cuboidal, columnar, pseudostratified, or ciliated epithelium.^11^

Surgical excision remains the treatment of choice in EDCs.^12^ This procedure should be undertaken as soon as possible in children diagnosed with an EDC, because they are expected to develop symptoms if they have not done so already.^13^ The number of studies supporting the thoracoscopic approach has increased quickly. Most of them present cohorts of patients with foregut duplication cysts, which are divided into groups of patients with EDCs and bronchogenic cysts after surgery.^14,15^ The biggest study that we found was performed by Bratau et al^15^, published in 2005. This research compared 16 cases of thoracotomy and 11 cases of thoracoscopy undertaken on children with foregut duplication cysts. Thirteen cases of EDCs were finally recognized in this cohort. Bratau et al observed a shorter duration of drainage and length of stay in cases of thoracoscopy. These authors also noted a lower incidence of intraoperative complications such as tracheal and esophageal injury during thoracoscopic excision of the mass. Nowadays, it is commonly said that video-assisted thoracoscopy reduces postoperative pain, shortens the recovery period and allows early hospital discharge compared with open surgical resection via thoracotomy. The cosmetic outcome with minimal skin scarring is undoubtedly another advantage of this method.^11^ The age of children who have undergone thoracoscopy is continually decreasing. There are some cases of infants diagnosed with an EDC and treated by thoracoscopy reported in the literature. The course of surgical treatment is supported by the ongoing development of pediatric endosurgical instruments.^16^

We have performed a successful thoracoscopic EDC excision in a newborn on the 15th day after birth. We decided to operate at this time because of the size (35 mm) of the mediastinal mass, which was expected to cause symptoms and significant lung compression in the short term. The patient was diagnosed with a mediastinal cyst during a prenatal ultrasound evaluation and the CT chest scan after birth confirmed this finding. This early diagnosis enabled early surgical intervention. The tolerance to thoracoscopy procedure in our patient was excellent. The postoperative period was uneventful, and the newborn was discharged within 1 week after surgery. One year of follow-up revealed no symptoms or recurrences (chest radiograph was performed). The histological examination confirmed the diagnosis of a foregut cyst. Based on these data and clinical findings, a final diagnosis of and EDC was established. Searching the literature (PubMed) using the MeSH terms esophageal, duplication, newborn, child, and thoracoscopy, we found only a few cases of such an early thoracoscopic intervention in the case of an EDC.

## CONCLUSIONS

EDCs are uncommon congenital anomalies. Their early diagnosis and minimally invasive excision via thoracoscopy seems to provide a good outcome. This approach should be considered as the treatment of choice for EDCs.
